# Eczema herpeticum subsequent to septic shock in early pregnancy: a first case report

**DOI:** 10.1186/s12879-021-06924-9

**Published:** 2021-12-14

**Authors:** Kiichiro Furuya, Yuki Takemoto, Hiroki Kurahashi, Harue Hayashida, Sho Fujiwara, Saya Yamashita, Yangsil Chang, Hiroaki Tsubouchi, Kayoko Shikado, Kazuhide Ogita

**Affiliations:** 1 Department of Obstetrics and Gynaecology, Rinku General Medical Centre, 2-23, Rinku Ourai-Kita, Izumisano, Osaka 598-8577 Japan; 2 School of Biosciences, Sutton Bonington Campus, University of Nottingham, Sutton Bonington, Leicestershire, LE12 5RD UK

**Keywords:** Eczema herpeticum, Atopic dermatitis, Pregnancy, Sepsis, Immunosuppression, Case report

## Abstract

**Background:**

Eczema herpeticum (EH) is a severe skin complication caused by human simplex virus (HSV) infection concomitant with immune dysfunction and dermatological conditions, mainly atopic dermatitis. We present the first case of EH subsequent to sepsis-related immunological suppression in pregnancy.

**Case presentation:**

Septic shock developed in a 30-year-old primiparous woman at 14 weeks of pregnancy during admission for hyperemesis gravidarum. Although her life-threatening status due to sepsis improved by prompt treatment, on day 3 of treatment in the intensive care unit, blisters suddenly erupted on her face and neck and spread over her body. EH was diagnosed according to HSV type-1 antigen positivity and a past medical history of EH and atopic dermatitis. Antiviral agents were administered immediately, with positive results. Her general condition improved quickly, without central nervous system defects. This is the first report of EH following septic shock in early pregnancy. At present, we speculate that EH develops as a complication due to immunological changes in the late phase of sepsis because sepsis is mainly characterized by both an inflammatory state in the acute phase and an immunosuppressive state in the late phase. Pregnancy can also contribute to its pathogenesis, as it causes an immunosuppressive state. Mortality due to EH is relatively high; in this case, a history of EH and atopic dermatitis contributed to the initiation of prompt medical interventions for the former, with improvement in the patient’s severe condition. The combination of immunological changes in sepsis and pregnancy can cause HSV reactivation, resulting in EH recurrence.

**Conclusions:**

In conclusion, if dermatological symptoms develop in a pregnant woman with a history of EH and/or atopic dermatitis treated for sepsis, EH should be suspected based not only on clinical features but also on immunological changes along with sepsis, and prompt medical interventions should be initiated.

## Background

Eczema herpeticum (EH) is a severe skin-related complication of herpes simplex virus (HSV) type 1 or 2 infection that is also known as Kaposi’s varicelliform eruption (KVE). EH is characterized by cutaneous pain and new vesicular skin lesions secondary to HSV type-1 or -2 infection. Although it is believed that the risks of EH are immunosuppression and some degree of epidermal barrier compromise due to dermatological problems, especially atopic disease, the definitive pathogenesis remains unknown [1]. We retrieved seven reports of EH in pregnancy from PubMed [2–8]; however, there is no reported case of EH subsequent to septic shock in pregnancy. Here, we present the first case of EH subsequent to bacterial septic shock in the 1st trimester of pregnancy.

## Case presentation

A 30-year-old primiparous Japanese woman at 14 weeks of pregnancy was admitted for hyperemesis gravidarum. She had a past history of atopic dermatitis and EH while not pregnant. At 14 weeks and 5 days of gestation, her condition suddenly worsened, with fever (> 38.0 °C), tachycardia (> 130 beats/min), tachypnoea (> 30 times/min) and vital shock (74/40 mmHg). Blood examination revealed an increased white blood cell count (> 9000/µL), neutrophil percentage (≒ 90%), and serum C-reactive protein (CRP) level (2.82 mg/dL). In addition to these increases indicating an inflammatory response, her serum procalcitonin level was extremely high (12.8 ng/mL). Arterial blood gas analysis showed hyperlactataemia. Tests for influenza type A/B, coronavirus disease 2019 (COVID-19), group A haemolytic streptococcus (*Streptococcus pyogenes*), and toxoplasmosis, other agents, rubella, cytomegalovirus, and herpes simplex (TORCH)-related virus infections were negative. Urine, vaginal discharge, blood, and nasal mucous samples were cultured. As her clinical symptoms, abnormal vital signs, increasing inflammatory response, and extremely high level of procalcitonin were indicative of septic shock, we transferred the patient to the intensive care unit (ICU) for close monitoring. Empirical antibiotic treatment and intravenous infusion loading were administered immediately. Despite no evident infectious lesions or foci on contrast-enhanced computed tomography (CT), apparent evidence of bacterial uterine infections with gram-positive rod bacteria and *Klebsiella aerogenes* was found by vaginal discharge culture. As a result, a clinical diagnosis of septic shock due to bacterial intrauterine infection was made.

After receiving prompt treatments for her life-threatening conditions and severe infection, her vital signs, including body temperature, blood pressure, and heart rate, rapidly returned to parameter values within normal ranges on day 3. According to laboratory data, indicators of an inflammatory response, including increased CRP and white blood cell count, also improved. She was discharged from the ICU and returned to the obstetrics ward.

**Fig. 1:**
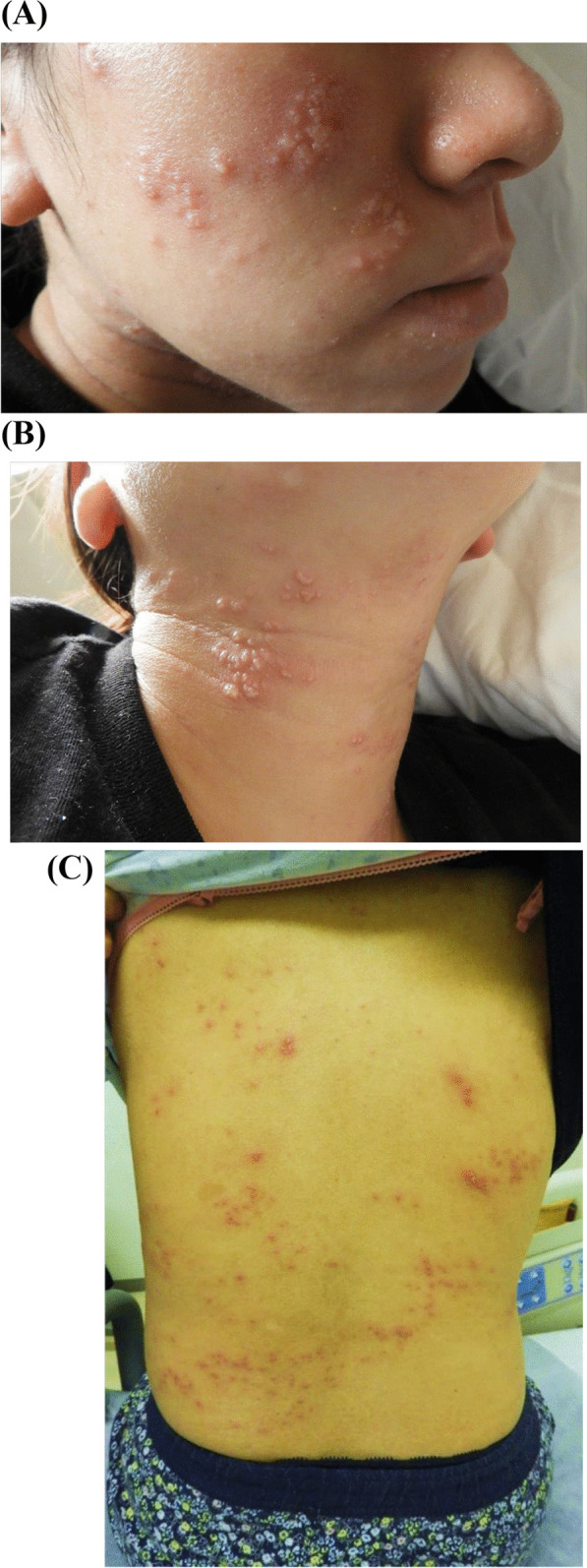
Skin eruptions and rash on the face (**A**), neck (**B**), and body (**C**) of a pregnant woman at 14 weeks and 6 days of gestation subsequent to septic shock

**Fig. 2:**
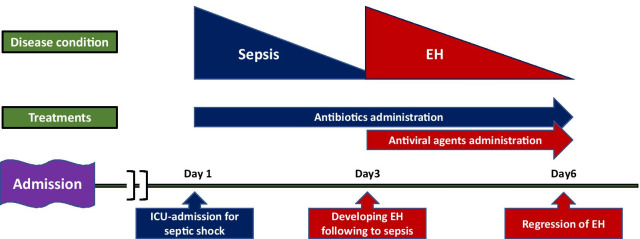
Disease conditions, treatments, and clinical time course of septic shock and eczema herpeticum. *EH* eczema herpeticum, *ICU* intensive care unit

However, rash-like eruptions on her face and neck suddenly emerged on the same day. The blisters spread rapidly and systemically within several hours (Fig. 1A–C). Due to her past medical history of severe EH and atopic dermatitis during nonpregnancy, we diagnosed EH recurrence and consulted dermatological experts. We administered oral acyclovir as systemic antiviral treatment. HSV type-1 antigen was detected in a scraping sample from a skin lesion, leading to a definitive diagnosis of EH. The patient’s skin condition improved after 3 days of acyclovir administration (Fig. 2). The blisters and eruptions had turned scaly, and her skin pain had decreased. Finally, she was discharged from our hospital without neurological complications and completed her prenatal check-ups at our outpatient clinic.

## Discussion and conclusions

We report the first case of EH in the 1st trimester of pregnancy subsequent to septic shock.

This report reveals three important findings relevant to daily obstetrical care.

First, we found that a patient’s dermatological background, mainly atopic dermatitis, plays a role in skin complications due to reactivation of HSV. EH is a severe HSV-related complication with possibly severe manifestations, including burn-like skin disorders and central nervous system dysfunction. One of the greatest risk factors of EH is a history of dermatological conditions such as atopic dermatitis, which compromises skin barrier function, and HSV infection [9]. Although there are few reports on EH in pregnancy to date, the status of pregnancy combined with a history of dermatological conditions appears to have the same risk as immunosuppression combined with a history of dermatological conditions, which are risk factors for EH [4]. Moreover, in this case, information on past medical history of atopic dermatitis and previous EH helped us to diagnose this dermatological problem as EH and immediately start anti-viral treatment before progression of her fatal condition.

Second, we found that women with a medical history of EH or atopic dermatitis may be vulnerable to EH recurrence, even in pregnancy. Several reports have indicated that patients with atopic dermatitis and immunological dysfunction or suppression, including pregnancy, are vulnerable to EH development or recurrence [4]. However, there have been few reports on EH in pregnant women with the same dermatologic problems and a medical history of HSV infection [2–8]; thus, its pathogenesis has remained unclear. The maternal immune system enters a suppressive state during pregnancy to tolerate the foetus, which contains genetic material from both the mother and father [10], and this immunosuppressive effect may play a role in EH development.

Finally, we suspect that immunosuppressive alterations in the post-acute stage of sepsis played a role in HSV reactivation in this case. According to previous studies, the immunological response in sepsis consists of two stages. The immune response during the acute phase of sepsis is characterized by inflammation and stimulation of the immune system, including the release of robust proinflammatory cytokines, an increase in phagocytes, and activation of killer T-cells, to eliminate pathogens. During the late phase of sepsis (2–3 days after onset), the immune system mobilizes anti-inflammatory factors to repair and heal tissues and cells damaged by inflammation. However, these alterations in immunosuppressive status may lead to a fatal condition due to secondary infection and reactivation of viral antigens [11–13]. In this case, EH developed rapidly after recovery from septic shock. This phenomenon can be explained by secondary reactivation of HSV due to post-sepsis immunosuppression. Some post-sepsis features of immunological dysfunction are as follows: (1) quantitative and qualitative defects in antigen-presenting cells (monocytes and dendritic cells), (2) alterations in T/B lymphocyte- and natural killer cell-mediated immunity, (3) relative increases in T-regulatory cells (T-regs), (4) decreases in gamma-globulin, (5) quantitative and qualitative defects in neutrophils, (6) increases in immature forms of neutrophils, and (7) hyper-/hypocytokinaemia. In particular, a decreasing number of natural killer cells results in reduced interferon-gamma (IFN-γ) and viral reactivation [13]. Unfortunately, in our institute, there was at that time no instrumentation, such as a flow cytometer, for detecting profiles of T cells, B cells, and natural killer cells, and we could not analyse these profiles. Nevertheless, from an observational point of view, the clinical course of the development of EH following sepsis in this case was compatible with the immunological changes due to the late phase of sepsis from previous reports, as described above.

In conclusion, we present the first case of EH subsequent to possible immunotolerance during pregnancy and immunological changes along with the late phase of sepsis in a patient with a history of dermatological conditions, including atopic dermatitis. Obstetricians should be aware of immunosuppression-related dermatological diseases, including EH, when a pregnant woman with dermatological backgrounds, especially atopic dermatitis and/or sepsis, exhibits symptoms indicative of fatal immunosuppressive effects.

## Data Availability

The data and materials are available on request from the corresponding author.

## Abbreviations

COVID-19: Coronavirus disease 2019
CRP: C-reactive protein
CT: Computed tomography
EH: Eczema herpeticum
HSV: Herpes simplex virus
ICU: Intensive care unit
IFN-γ: Interferon-gamma
KVE: Kaposi’s varicelliform eruption
TORCH: Toxoplasmosis, other agents, rubella, cytomegalovirus, and herpes simplex
T-regs: T-regulatory cells
